# Alveolar rhabdomyosarcoma – The molecular drivers of PAX3/7-FOXO1-induced tumorigenesis

**DOI:** 10.1186/2044-5040-2-25

**Published:** 2012-12-03

**Authors:** Amy D Marshall, Gerard C Grosveld

**Affiliations:** 1 Department of Genetics, St Jude Children’s Research Hospital, Memphis, TN, 38105, USA; 2 Gene and Stem Cell Therapy Laboratory, Centenary Institute, University of Sydney, Missenden Road, Camperdown, NSW, 2050, Australia

**Keywords:** Alveolar rhabdomyosarcoma, PAX3-FOXO1, PAX7-FOXO1, FGFR4, CNR1, IRIZIO, N-MYC, IGF2, MET, CXCR4, p53, MDM2, P-Cadherin, TFAP2B, miR17-92

## Abstract

Rhabdomyosarcoma is a soft tissue sarcoma arising from cells of a mesenchymal or skeletal muscle lineage. Alveolar rhabdomyosarcoma (ARMS) is more aggressive than the more common embryonal (ERMS) subtype. ARMS is more prone to metastasis and carries a poorer prognosis. In contrast to ERMS, the majority of ARMS tumors carry one of several characteristic chromosomal translocations, such as t(2;13)(q35;q14), which results in the expression of a PAX3-FOXO1 fusion transcription factor. In this review we discuss the genes that cooperate with PAX3-FOXO1, as well as the target genes of the fusion transcription factor that contribute to various aspects of ARMS tumorigenesis. The characterization of these pathways will lead to a better understanding of ARMS tumorigenesis and will allow the design of novel targeted therapies that will lead to better treatment for this aggressive pediatric tumor.

## Introduction

According to the American Cancer Society, rhabdomyosarcoma (RMS) comprises about three percent of childhood cancers, with about 350 new cases occurring annually in the US [1], and it affects slightly more males than females [2]. RMS is a small, round, blue cell tumor usually arising in skeletal muscle tissue, and it is thought to originate from mesenchymal cells likely committed to the skeletal muscle lineage. Consistent with a myogenic origin, RMS tumors express skeletal muscle markers such as skeletal muscle actin and myosin, desmin, myoglobin, Z-band protein, MYOD and often myogenin [3-7]. RMS consists of two major histological subtypes, embryonal and alveolar RMS. The embryonal subtype (ERMS) is thought to be histologically reminiscent of embryonic developing skeletal muscle [7]. ERMS is the most prevalent of the subtypes, accounting for about 60% of RMS cases [2]. It occurs mainly in children younger than 10 years and is usually associated with a favorable prognosis, with a failure-free survival rate of 81% [8,9]. Tumors usually localize to the head and neck (including the extraocular muscles of the eye), the genitourinary tract and the retroperitoneum [7,8]. The alveolar subtype (ARMS) constitutes approximately another 20% of RMS cases [10] and occurs predominantly in adolescents. Histologically, ARMS tumors typically contain alveoli structures similar in appearance to those seen in the lung [7], though solid-variant ARMS does occur [11]. Primary ARMS tumors typically arise in the extremities and trunk [7-9,12], and they are more aggressive than their ERMS counterparts. ARMS is associated with a poorer prognosis, with a 5-year failure-free survival of 65% [8].

**Figure 1:**
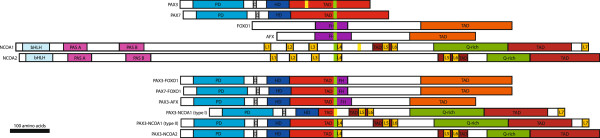
**Gene translocation in alveolar rhabdomyosarcoma. **Scale diagram showing the parent proteins and the resulting fusion proteins arising from chromosomal translocations occurring in ARMS. Green or yellow indicates the protein fusion sites [14,19-22]. Homologous domains are indicated in like colors. DNA-binding domains are indicated as: paired domain (PD), homeodomain (HD), fork head DNA-binding domain (FH) and basic helix-loop-helix domain (bHLH). Regions of the proteins known to act as transcriptional activation domains are indicated (TAD). Other domains include the octapeptide domain (O), PAS domains (PAS A/B), LXXLL motifs (L1-L7) and glutamine-rich region (Q-rich). Maps were derived from the following references: [14,19-22,32-34].

A characteristic of the ARMS subtype is the occurrence of recurrent chromosomal translocations. The most common of these is t(2;13)(q35;q14), which results in the expression of an oncogenic fusion protein. This fusion protein consists of the paired and homeodomains of the PAX3 transcription factor with the potent transcriptional activation domain of FOXO1 (FKHR), a member of the forkhead (FOX) family of transcription factors [13-15]. The PAX3 homeodomain is required to recapitulate PAX3-FOXO1-induced tumorigenesis, though the paired domain may play a minor role [16,17]. The PAX3-FOXO1 fusion protein can be detected in about 55% of ARMS cases [18]. A similar translocation of t(1;13)(p36;q14) fuses the PAX7 DNA-binding domains, the closest homolog of PAX3, to FOXO1 [19]. This translocation occurs in a further 22% of ARMS cases [18]. Recently, further similar translocations have been found in individual ARMS cases: t(2;X)(q35;q13), which results in PAX3-AFX fusion [20], and t(2;2)(q35;p23) and t(2;8)(q35;q13), which generate a fusion protein of PAX3-NCOA1 and PAX3-NCOA2, respectively [21,22]. These “cryptic” rare fusion variants are thought to be present in up to another 10% of ARMS tumors [7]. During normal development, PAX3 expression occurs in the neural tube and dermomyotome [23], and it is required for the normal migration of skeletal muscle precursors to the limb bud [24]. PAX7 expression is a marker of satellite cells in adult skeletal muscle [25] and is required for normal self-renewal [26]. Unlike skeletal muscle-specific PAX3 and PAX7, FOXO1A, AFX, NCOA1 and NCOA2 are widely expressed and mediate gene transcription downstream of cell signaling pathways [27-31]. All of the ARMS fusion proteins consist of the PAX3/7 DNA-binding domains fused to the transcriptional activation domains of more potent transcription factors (see Figure 1) [14,15,22]. Genome-wide transcription factor-binding studies have not yet been performed to determine whether wild-type PAX3 and PAX7-binding sites differ from these PAX3/7 fusion transcription factor-binding sites.

Expression of these ARMS fusion transcription factors is thought to abrogate normal skeletal muscle differentiation, allowing aberrant cell division and tumor development. PAX3 expression can inhibit myogenic differentiation of cultured myoblasts [35]. Although PAX3 protein is rapidly degraded during early myogenic differentiation, PAX3-FOXO1 has a significantly longer half-life than wild-type PAX3 [36]. PAX3/7-FOXO1 is capable of suppressing MyoD expression and activity [37,38]. PAX7-FOXO1 expression induces NFκB signaling, which inhibits myogenesis via activation of cyclin D1/CDK4 complexes. These complexes sequester MyoD, which would normally drive cell cycle withdrawal and myogenic differentiation [39]. In addition transcriptionally inactive MyoD can enhance PAX3-FOXO1 transcriptional activity [37]. There is also evidence that MyoD transcriptional activity is abrogated in ERMS tumors [40].

The remaining ARMS tumors are classed as fusion-negative ARMS. However, fusion-negative ARMS are indistinguishable on the levels of gene expression and in clinical outcome from ERMS tumors, leading some to argue that translocation status should be the defining factor of ARMS [41-44]. Within the ARMS subtype, prognosis can vary by disease stage at diagnosis as well as translocation status. For example, patients presenting with metastatic disease have an estimated 4-year overall survival rate of 75% for PAX7-FOXO1, while those with the PAX3-FOXO1 translocation have only 8% estimated 4-year overall survival [18]. Indeed, there is evidence that the PAX3-FOXO1 is a more potent oncogene than PAX7-FOXO1. Barr *et al.*[45] found that only 1/24 PAX3-FOXO1-positive ARMS tumors had amplification of the PAX3-FOXO1 gene, while PAX7-FOXO1 was amplified in 10/11 PAX7-FOXO1 ARMS, implying that genomic amplification of PAX7-FOXO1 is required for tumorigenesis, while a single copy of PAX3-FOXO1 is sufficient. However the gene expression profiles of PAX3-FOXO1- and PAX7-FOXO1-expressing tumors have not, to the author’s knowledge, been specifically compared to identify the gene set responsible for this difference in prognosis between ARMS tumors with these two fusion genes.

## Review

PAX3-FOXO1 is the most common fusion gene in ARMS. This fusion transcription factor is thought to drive the gene expression that causes the worse prognosis in ARMS tumors. Many studies have sought to identify the differences in gene expression between ERMS and ARMS, as well as the genes aberrantly regulated by PAX3-FOXO1. In this review, we summarize these gene expression changes specific to PAX3-FOXO1 expression and/or ARMS. Moreover, we consolidate these data into a list of genes that may well represent the means by which ARMS tumors obtain a more aggressive phenotype than ERMS.

### Cooperating mutations in ARMS tumors

It is likely that PAX3/7-FOXO1 translocation is one of the earliest events in ARMS tumorigenesis as it occurs in the majority of ARMS cases, more often than any other genetic lesion characterized in the disease. However, PAX3-FOXO1 expression in normal cells is not sufficient to induce transformation, and other genetic alterations are required [46-48]. Genomic amplification is common in ARMS tumors. The three most common amplifications seen in ARMS involve regions of chromosomes 2, 12 and 13 [49].

The region of chromosome 12 amplification spans 12q13-15 and is reported in 28% to 56% of ARMS tumors [49-52]. This 12q13-15 region includes genes such as C/EBP-homolog and transcription factor CHOP/DDIT3/GADD153, sarcoma-amplified sequence and transmembrane 4 superfamily member SAS/TSPAN31, alpha 2-macroglobulin receptor A2MR/LRP1, Sonic hedgehog (SHH) pathway effector and zinc finger transcription factor GLI1, cyclin-dependent kinase cell cycle regulator CDK4 and p53 pathway modulator MDM2. In most cases, gene amplification accompanies an increase in gene expression [50,53].

Though GLI1 is amplified genetically, the expression of this gene is not always associated with its genetic amplification. When GLI1 is overexpressed in RMS, it has been associated with an undifferentiated subtype rather than ERMS or ARMS, indicating that GLI1 may play a role in tumors that show primitive histopathological features [54]. Thus, GLI1 overexpression cannot be well associated with the ARMS pathology.

MDM2 is perhaps the best candidate oncogene in this region because of its inhibitory effect on p53 function [55]. However, MDM2 is not always included in this 12q13-15 amplification. RH30, an ARMS cell line, lacks amplification of MDM2 but shows amplification and overexpression of SAS, CHOP, GLI1 and A2MR [53]. In addition, the frequency of MDM2 gene amplification specifically may be as low as 10% in ARMS tumors [56]. One study found only 2 of 34 ARMS samples to be highly immunoreactive for MDM2 [12]. Moreover, MDM2 expression shows no association with patient prognosis or other clinicopathologic parameters [12]. Thus, it may be amplification of one of the other genes at this chromosome 12 locus that is the important cooperating mutation with PAX3-FOXO1.

Other alterations in the p53 pathway have been found in ARMS. In ARMS tumor samples mutated p53 was reported in 0 to 22% of cases [12,56,57]. RMS cell lines show a significantly higher rate of p53 abnormalities with 60%, indicating establishment of these cell lines through xenograft and cell culture increases the proportion of cell lines with p53 alterations [56]. Looking at p53 and MDM2 expression levels, both are low in ARMS and ERMS. Metastatic ERMS tumors show significantly higher p53 protein expression, indicating that p53 gene alterations are a late event in rhabdomyosarcomagenesis. Again, p53 status did not show any correlation to prognosis [58].

Chromosome 2 has been shown to be amplified at 2p24 in 32 to 60% of ARMS tumors [49,51,59,60]. This region is known to contain the proto-oncogene N-MYC. Two independent studies have shown that a gain in the genomic copy number of the N-MYC gene is associated with an unfavorable disease outcome [59,61]. In addition, N-MYC is more highly expressed in ARMS cells lines than ERMS lines, despite the fact that it was found to only be genomically amplified in one of the five lines, indicating more than one mechanism of N-MYC overexpression in ARMS. However, in this study no clear relationship in N-MYC expression was seen with regard to primary tumor samples [62].

Another chromosomal region frequently amplified in ARMS is 13q31-32, showing amplification in between 14 and 19% of ARMS tumors [49,51]. Presence of this amplification is significantly associated with poorer failure-free survival in ARMS [63]. The minimum overlapping region of amplification at this region was originally defined as only containing two genes: GPC5 and C13ORF25. The C12ORF25 gene encodes the micro-RNA cluster miR-17-92 (MIR17HG) in an intron [64]. GPC5 overexpression can increase cell proliferation through the modulation of the growth factor activity of FGF2, HGF and WNT1a [64]. However, more thorough mapping of the genetic amplification showed that the entire GPC5 locus was only amplified in 12.5% of 13q31 amplified ARMS tumors, while the minimally amplified region contains only the peptidylprolyl isomerase pseudogene (LOC390419) and MIR17HG. This amplification is particularly prevalent in PAX7-FOXO1-positive ARMS tumors. The miR-17-92 cluster of micro-RNAs has been shown to play a role in a variety of cancer types (for review, see [65]). In PAX7-FOXO1, but not PAX3-FOXO1 expressing ARMS, overexpression of miR-17, -19a, -19b, 20a and 92a is specifically associated with an increased rate of 2-year treatment failure. This indicates a possible pro-tumorigenic interaction between PAX7-FOXO1 and miR-17-92 locus overexpression [63].

Rhabdomyosarcoma can also be associated with a loss of heterozygosity (LOH) or loss of imprinting (LOI) at 11p15.5 [66,67]. This region contains several imprinted genes such as IGF2, which is maternally imprinted (paternal allele is expressed), and H19 and p57/Kip2, which are paternally imprinted (maternal allele is expressed) [68-70]. IGF2 expression appears to be specifically upregulated by changes in imprinting or LOH at this locus in RMS. ERMS tumors are associated predominantly with a LOH at the IGF2 locus, though there is some discrepancy in the proportion of ERMS tumors showing this change: 23% according to Anderson *et al*. [66] and 72% according to Visser *et al*. [67]. Conversely, IGF2 is upregulated by LOI in 46% of fusion-positive ARMS tumors, while imprinting of H19 is conserved in 93% [66]. This indicates that an increase in IGF2 expression in RMS is important for tumorigenesis, though the mechanism of this upregulation, either LOH or LOI, varies by subtype.

A screen for PAX3-FOXO1-interacting proteins using ARF−/− primary mouse myoblasts expressing PAX3-FOXO1 and an RH30 cDNA expression library identified a gene that could induce tumor formation where ARF−/− myoblasts expressing PAX3-FOXO1 alone did not. The RH30 gene expression library expressed a truncated fragment of this novel gene dubbed IRIZIO, and expression of either this truncated form or the full-length wild-type IRIZIO were protumorigenic in this model [71]. Due to the nature of the screen, and given that abrogation of the p53 and pRb pathways are required for PAX3-FOXO1-driven cell transformation [46,47], this screen was expected to identify proteins that could abrogate the pRb pathway [71]. The mechanism of the interaction between IRIZIO and pRb, however, has yet to be identified.

### PAX3-FOXO1 target genes

Many gene expression studies have been performed by various groups to try to identify genes that are either downstream of PAX3-FOXO1 gene expression in various cell types or are indicative of ARMS tumor gene expression profiles (see Table 1). Only a small proportion of these studies have gone on to further investigate the mechanism of PAX3-FOXO1 regulation of these genes and/or what role these genes may play in ARMS tumorigenesis.

**Table 1:** PAX3-FOXO1 target genes in ARMS

**Gene**	**Description**	**Regulation**	**References**	**PF target**
ABAT	4-Aminobutyrate aminotransferase;	Up	[72-74]	Yes
ACTC	Actin, alpha, cardiac muscle 1	Up	[5,75]	Yes
ADAM10	A disintegrin and metalloproteinase domain 10	Up	[73][72]	Yes
ADRA2A	Alpha-2 adrenergic receptor subtype C10	Up	[72,74,76]	Yes
ADRA2C	Alpha-2 adrenergic receptor subtype C4	Up	[22,74,76]	Yes
ALK	ALK tyrosine kinase receptor, anaplastic lymphoma kinase Ki-1	Up	[22,72,74,76,77]	Yes
ANK2	Ankyrin 2	Up	[73,74,76]	Yes
ASS	Argininosuccinate synthase	Up	[73,74,78]	?
ASTN2	Astrotactin 2	Up	[72,76]	Yes
BIN1	Bridging integrator 1	Down	[72,79]	Yes
BMP5	Bone morphogenic protein 5	Up	[72-74,76]	Yes
C10ORF6	Family with sequence similarity 178, member A	Up	[75,76]	Yes
CCND1	Cyclin D1	Down	[75][79]	?
CD9	CD9 molecule/Tetraspanin-29	Up	[73,76]	Yes
**CDH3**	Cadherin 3, type 1, **P**-**cadherin** (placental)	Up	[72,76,80]	Yes
CHD7	Chromodomain helicase DNA-binding protein 7	Up	[76,81]	Yes
CKM	Creatine kinase M chain, muscle	Up	[5,79]	Yes
**CNR1**	Cannabinoid receptor 1	Up	[22,72,74,82-84]	Yes
COL18A1	Collagen type 18 α 1	Up	[73,74]	?
**CXCR4**	C-X-C chemokine receptor type 4	Up	[75,76,85-87]	Yes
CXCR7	C-X-C chemokine receptor type 7	Down	[76,87]	Yes
DCX	Neuronal migration protein doublecortin	Up	[73,74,81]	?
DES	Desmin	Down	[72,79]	Yes
DKFZP762M127	Unknown	Up	[73,74,81]	Yes
DUSP4	Dual specificity phosphatase 4	Down	[73,81]	?
DZIP3	DAZ interacting protein 3, zinc finger	Up	[72,74,81]	Yes
ELA1	Elastase-1	Up	[22,72,74]	Yes
ENC1	Ectodermal-neural cortex 1 (with BTB-like domain)	Up	[75,76]	Yes
ENO3	Enolase 3 (beta, muscle)	Up	[74,79]	?
EPHA4	Ephrin type-A receptor 4	Up/down	[76,81,82]	Yes
FGFR2	Fibroblast growth factor receptor 2	Up	[72,76]	Yes
**FGFR4**	Fibroblast growth factor receptor 4	Up	[22,73,76,88,89]	Yes
FLNB	Filamin B, beta	Down	[76,82]	Yes
FNBP1	Formin-binding protein 1	Down	[22,75,76]	?
FOXF1	Forkhead box protein F1	Up	[22,72,74,76,81]	Yes
FOXO1	Forkhead box O1	Up	[75,76]	Yes
GADD45A	Growth arrest and DNA-damage-inducible protein GADD45 alpha	Up	[73,76,81]	Yes
GRAF	GTPase regulator associated with FAK	Up	[22,72]	Yes
GTF3C1	General transcription factor 3C	Up	[72,81]	Yes
**H19**	Imprinted maternally expressed gene, untranslated mRNA	Up	[5,79]	Yes
HDAC5	Histone deacetylase 5	Up	[73,76]	Yes
HUMMLC2B	Myosin regulatory light chain 2, skeletal muscle isoform	Down	[72,73]	Yes
**IGF2**	Insulin-like growth factor II	Up	[5,75]	Yes
IGFBP3	IGF-binding protein 3	Down	[73,81]	Yes
IGFBP5	IGF-binding protein 5	Up	[5]	Yes
IL4R	Interleukin 4 receptor	Up	[72,73,75,76]	Yes
JAKMIP2	Janus kinase and microtubule-interacting protein 2	Up	[72-74,76]	Yes
KCNN3	Small conductance calcium-activated potassium channel protein 3	Up	[73,74,81]	Yes
KCNS3	Potassium voltage-gated channel subfamily S member 3	Up	[73,74]	?
LRRFIP2	Leucine-rich repeat (in FLII) interacting protein 2	Up	[72,74]	Yes
MARCH3	Membrane-associated RING finger protein 3	Up	[73,81]	Yes
MCAM	Melanoma cell adhesion molecule	Up	[73,81]	Yes
MEG3	Maternally expressed 3	Up	[73-75]	?
MN1	Meningioma (disrupted in balanced translocation) 1	Up	[73,76]	Yes
**MET**	Hepatocyte growth factor receptor	Up	[73,76,81,90-92]	Yes
MTUS2	Microtubule associated tumor suppressor candidate 2	Up	[72,76]	Yes
MYBPH	Myosin-binding protein H	Up/down	[5,72,75]	Yes
**MYCN**	N-MYC proto-oncogene protein	Up	[72,73,76,78,81]	Yes
MYH8	Myosin, heavy chain 8, skeletal muscle, perinatal	Up/down	[5,72]	Yes
MYL1	Myosin, light chain 1, alkali; skeletal, fast	Up/down	[5,79]	Yes
MYL4	Myosin, light chain 4, alkali; atrial, embryonic	Up/down	[5,72]	Yes
MYOD	Myoblast determination protein 1	Up	[5,73,76,81]	Yes
MYOG	Myogenin (myogenic factor 4)	Up	[5,90]	Yes
NEBL	Nebulette	Up/down	[72,73,76,81]	Yes
NELL1	NEL-like protein 1	Up	[22,72-74,76]	Yes
NHLH1	Nescient helix loop helix 1	Up	[22,76]	Yes
NPTX2	Neuronal pentraxin II	Down	[76,81]	Yes
NRCAM	Neuronal cell adhesion molecule	Up	[72-74]	Yes
OLIG2	Oligodendrocyte transcription factor 2	Up	[22,74]	?
PALMD	Palmdelphin	Down	[72,76]	Yes
PBK	PDZ-binding kinase	Up	[73,76]	Yes
PCDH7	Protocadherin 7	Up	[76,82]	Yes
PDZRN3	PDZ domain containing ring finger 3	Up	[74,76]	Yes
PGBD5	PiggyBac transposable element-derived protein 5	Up	[22,72,74]	Yes
PHF17	PHD finger protein 17	Up	[74,76]	Yes
PIPOX	Pipecolic acid oxidase	Up	[22,72,74,76]	Yes
PKP1	Plakophilin 1 (ectodermal dysplasia/skin fragility syndrome)	Up	[72,76]	Yes
PLAG1	Pleiomorphic adenoma gene 1 protein	Down	[75,81]	Yes
PLK2	Polo-like kinase 2	Down	[73,76]	Yes
PODXL	Podocalyxin-like protein 1	Up	[22,74,76]	Yes
POU4F1	Brain-specific homeobox/POU domain protein 3A	Up	[72,73,76]	Yes
PPARGC1A	Peroxisome proliferator-activated receptor gamma, coactivator 1 alpha	Up	[22,76]	Yes
PRKAR2B	Protein kinase, cAMP-dependent, regulatory, type II, beta	Up	[73,81,82]	Yes
PRKCA	Protein kinase C, alpha	Up	[73,76]	Yes
PSEN2	Presenilin 2 (Alzheimer’s disease 4)	Up	[73,74]	?
PTHLT	Parathyroid hormone-like hormone	Up	[76,82]	Yes
QDPR	Quinoid dihydropteridine reductase	Up	[74,81]	Yes
RASSF4	Ras association (RalGDS/AF-6) domain family 4	Up	[72,74,76]	Yes
RRP22	Ras-like protein family member 10A, on chm 22	Up	[73,74]	?
RYR1	Skeletal muscle-type ryanodine receptor	Up	[5,74]	Yes
RYR3	Brain-type ryanodine receptor	Up	[74,76,81]	Yes
SLC24A3	Solute carrier family 24 (sodium/potassium/calcium exchanger), member 3	Up	[74,76]	Yes
SIX1	SIX homeobox 1	Up	[5,75]	Yes
SOX14	SRY (sex determining region Y)-box 14	Up	[22,76]	Yes
STX11	Syntaxin 11	Up	[76,82]	Yes
SULF1	Sulfatase 1	Up	[73,76]	Yes
SVIL	Supervillin	Down	[72,76]	Yes
TCF712	Transcription factor 7-like 2 (T-cell specific, HMG-box)	Up	[73,81]	Yes
TGFB1	Transforming growth factor, beta 1	Up	[5,76]	Yes
**TFAP2B**	Transcription factor AP-2 beta	Up	[22,72]	Yes
TIAF1	TGF-beta-1-induced antiapoptotic factor 1	Up	[73,74]	?
TM4SF10	Transmembrane 4 superfamily member 10	Up	[73,81]	Yes
TNFAIP3	Tumor necrosis factor, alpha-induced protein 3	Up	[73,76]	Yes
TNNC2	Troponin C type 2 (fast)	Up/down	[5,72,73,79]	Yes
TNNI2	Troponin I type 2 (skeletal, fast)	Up/down	[5,72]	Yes
TNNT2	Troponin T type 2 (cardiac)	Up	[5,79]	Yes
TNNT3	Troponin T type 3 (skeletal, fast)	Down	[72,79]	Yes
TRAM2	Translocation-associated membrane protein 2	Up	[73,76]	Yes
TSC22D2	TSC22 domain family, member 2	Up	[74,76]	Yes
UBE2G2	Ubiquitin-conjugating enzyme E2G 2 (UBC7 homolog, yeast)	Up	[22,76]	Yes
WSCD1	WSC domain-containing protein 1	Up	[22,74]	?
WVA5A	Von Willebrand factor A domain containing 5A	Up	[72-74]	Yes

Summary of genes found to be differentially regulated in more than one reference in ARMS tumors and/or cell lines, or by PAX3-FOXO1 overexpression, in various cell types. Gene name and description are indicated and also the relative expression in ARMS or PAX3-FOXO1. Also indicated is whether the study indicates that these genes are downstream of PAX3-FOXO1 expression. Genes indicated in bold will be further discussed.

Two of these genes have already been mentioned as cooperating mutations seen in ARMS tumors, N-MYC and IGF2. The N-MYC locus is known to be amplified in a proportion of ARMS tumors, and the IGF2 locus is known to show LOI in ARMS tumors (see cooperating mutations in ARMS tumors). However, these studies also indicate that PAX3-FOXO1 may regulate the gene expression from these loci.

N-MYC expression has been shown to be upregulated in four independent studies using PAX3-FOXO1 targeting siRNA in the ARMS cell line, RH4 [72], PAX3-FOXO1 overexpression in the RD (ERMS) cell line [73,81] and in ARMS versus non ARMS tumor cell lines [78]. A tamoxifen (4-OHT)-inducible PAX3-FOXO1-ER construct induced upregulation of both N-MYC mRNA and protein in the transduced ERMS cell line, RD, and this was not sensitive to cycloheximide treatment, indicating that N-MYC is a direct transcriptional target of PAX3-FOXO1. However the PAX3-FOXO1 regulatory region for N-MYC did not appear to be contained within −1871 to +1058 of the N-MYC gene. Consistent with a transforming role for both PAX3-FOXO1 and N-MYC, the two genes synergized in soft agar colony-forming assays [81]. In addition, knockdown of N-MYC expression identified a positive feedback loop between N-MYC and PAX3-FOXO1 [93].

IGF2 was shown to be specifically overexpressed in ARMS compared to Ewing’s sarcoma cell lines [75], which is perhaps not surprising given that LOI of the IGF2 is seen in almost half of ARMS tumors [66]. However, Khan *et al*. [5] have shown that, in NIH3T3 cells, PAX3-FOXO1 expression can induce the upregulation of IGF2 mRNA. Interestingly, H19 expression was also found to be upregulated in response to PAX3-FOXO1 expression in these cells. Khan *et al*. [5] did not investigate whether PAX3-FOXO1 can regulate imprinting of this locus, and the mechanism of PAX3-FOXO1 regulation of IGF2 mRNA expression remains unknown.

Two factors shown to be upregulated in PAX3-FOXO1-expressing cells, MET [73,81,90,91] and CXCR4 [85,86], are thought to play a role in the increased propensity for metastasis seen with ARMS.

MET is the receptor for hepatocyte growth factor/scatter factor (HGF/SF) [94]. HGF-MET signaling has been shown to play an important role in both normal skeletal muscle development and regeneration, and it is involved in regulating myogenic cell migration, survival, proliferation and differentiation [95,96]. MET appears to be a downstream target of PAX3. Splotch mice, which express a mutant PAX3 and fail to form limb muscles because of an inhibition of myogenic precursor cell migration, show a decreased expression of MET [24,97]. MET has also been found in five independent studies to be downstream of the PAX3-FOXO1 fusion protein in ARMS [73,81,90-92]. However, in the case of ARMS, it appears that HGF may play a role in chemoattraction of tumor cells to the bone marrow, which is a common site of metastasis in ARMS cases [98,99]. ARMS cell line CW9019 shows chemotaxis toward bone marrow-derived-fibroblast-conditioned media *in vitro*, and this migration is inhibited by the MET-blocking agent, K-252a. Moreover, both RH30 and CW9019 ARMS lines home to the bone marrow in lethally irradiated mice, while the ERMS cell lines RD, SMS-CTR, and RH18 do not [99]. Conversely, siRNA against PAX3-FOXO1 prevents migration of cells in wound-healing assays of RH30 cells in response to HGF. In addition to a migratory function for MET in ARMS, knockdown of MET by shRNA in ERMS and ARMS inhibits cell proliferation and induces apoptosis. Moreover, shRNA-mediated knockdown of MET inhibits anchorage-independent growth of ARMS and ERMS, and mutant MET-expressing MEFS prevent PAX3-FKHR transformation [91]. Consistent with MET as a PAX3-FOXO1 target, high MET expression in RMS correlates with ARMS histology, advanced disease at diagnosis and bone marrow involvement [100].

CXCR4 is normally expressed in satellite cells within skeletal muscle and is used as a marker of mononucleated cells capable of differentiating into myofibers [101]. CXCR4 is a cell surface receptor; it binds and mediates the signaling of stromal-derived factor-1 (SDF-1) and induces cell chemotaxis [102]. SDF-1 can induce migration and chemotactic invasion in ARMS cell lines, but not ERMS cell lines [85], and this migration can be inhibited by SDF-1-neutralizing antibody or the CXCR4 inhibitor AMD3100 [103]. Moreover, expression of CXCR4 and MET in ARMS lines appears to synergize to induce the migration of cells toward bone marrow-derived fibroblast conditioned media *in vitro*. Inhibition of each receptor reduces migration, and combined inhibition reveals synergism between these receptors [99]. SDF-1 can also induce proliferation of the ARMS cell line, RH30 [103]. Consistent with CXCR4 expression being downstream of PAX3-FOXO1 transgene expression, CXCR4 expression in RMS correlates with the ARMS histology, unfavorable primary site, advanced disease at diagnosis and bone marrow involvement [100].

Other genes have been further confirmed as downstream genes of PAX3-FOXO1. Cannabinoid receptor 1 (CNR1) is specifically upregulated at both the mRNA and protein level in fusion-positive ARMS cells [74]. CNR1 is normally highly expressed in brain [104] but is also expressed in skeletal muscle at levels detectable by RT-PCR [105]. CNR1 has been confirmed, using ChIP analysis, as a direct target of both PAX3 and PAX3-FOXO1 transcriptional activity [82]. Furthermore, the homeodomain of PAX3-FOXO1 appears to be the important domain for the regulation of CNR1 expression [72]. CNR1 has been proposed to be a potential drug target in ARMS. Treatment with CNR1 agonists can induce apoptosis in some ARMS cell lines [83]. In addition, CNR1 expression has been linked with an increase propensity for PAX3-FOXO1 expressing mouse myoblast invasiveness and lung metastasis formation. Moreover, treatment with an inverse agonist to CNR1 can abrogate *in vitro* invasion and *in vivo* lung metastasis formation [84]. Thus, CNR1 may represent a viable therapeutic target specific for the increased metastatic capacity of PAX3-FOXO1 expressing ARMS.

Transcription factor AP2β (TFAP2B) has been shown to be a downstream target of PAX3-FOXO1 and appears to require the paired domain of PAX3-FOXO1 to be induced. TFAP2B promoter expression is induced by PAX3, which has been shown to bind to the TFAP2B promoter by ChIP analysis. siRNA targeting TFAP2B introduced into PAX3-FKHR-positive ARMS induces apoptosis, indicating that TFAP2B can mediate cell survival in ARMS, downstream of PAX3-FOXO1 [72].

FGFR4 has been identified as a direct transcriptional target of PAX3 and PAX3-FOXO1, which bind to a downstream enhancer region [76,106]. Accordingly, FGFR4 is significantly upregulated by PAX3-FOXO1 expression [22,73,76,88,89,107]. However, upregulation of FGFR4 downstream of PAX3-FOXO1 in primary myoblasts does not appear to act as an effector of PAX3-FOXO1-mediated myoblast transformation given that wild-type FGFR4 upregulation is not required for PAX3-FOXO1-induced proliferation, transformation, invasion or inhibition of myogenic differentiation [89]. However, knockdown of FGFR4 in RMS cell lines does show a reduction in cell proliferation and an increase in apoptosis, suggesting that at later stages of ARMS tumorigenesis FGFR4 overexpression may interact with other unknown genetic lesions within these cell lines to induce pro-survival and proliferation effects [107]. It is interesting to note however that kinase domain-activating mutations in FGFR4 have been identified in 7.5% of RMS, including fusion-positive ARMS [108], and can contribute to myoblast growth advantage and transformation [89]. Thus, FGFR4-activating mutations likely represent cooperating mutations in RMS and upregulation of FGFR4 in fusion-positive ARMS would enhance this effect.

The CDH3/P-cadherin gene has been identified as a direct transcriptional target of PAX3/7-FOXO1 [72,76,80]. P-cadherin expression in the C2C12 myoblast cell line inhibits myogenic differentiation and maintains a proliferative state through maintaining cyclin D1 expression. This in turn results in transformation of C2C12 cells, allowing colony formation in soft agar. Additionally, P-cadherin expression resulted in enhanced cell motility, as well as cadherin switching, a hallmark of epithelial to mesenchymal transition and metastatic progression [80].

### *In vitro* models of ARMS

Many different cell lines have been derived from human ARMS tumors; these are regularly used to investigate the biology of ARMS. These include ARMS cell lines derived from human tumors in the laboratory of Dr. Peter Houghton: RH3, RH4, RH10, RH28, RH30 and RH41, all of which express the PAX3-FOXO1 fusion protein [13,109,110] and have been widely used in the field. In addition, the NCI-supported Pediatric Preclinical Testing Program (PPTP) uses RH10, RH28, RH30, RH30R, RH41 and RH65 subcutaneous xenograft tumors to test drug efficacy in a well-characterized preclinical model of many pediatric cancers [111-113].

Other *in vitro* models that have been used involve the introduction of the PAX3/7-FOXO1 fusion proteins into both myogenic and non-myogenic cell lines including fibroblast cell lines, ERMS cell lines, MEFs, mesenchymal stem cells (MSC), normal or immortalized human or mouse myogenic cells and even an osteosarcoma cell line [5,81,82,91,114-119]. Given that the cell of origin for ARMS has yet to be identified, perhaps this variation in model cell lines is prudent. However, it is likely that ARMS and ERMS tumors are derived from a mesenchymal cell likely of the the myogenic lineage because of skeletal muscle lineage-specific gene expression seen in these tumors [7].

Conversely, some studies have used endogenous PAX3-FOXO1 in ARMS cell lines to determine the transcriptional targets of this fusion protein within the ARMS tumor cell context. Both Kikuchi *et al*. [90] and Ebauer *et al*. [72] used siRNA specifically targeting PAX3-FOXO1 or both PAX3 and PAX3-FOXO1 sequences, respectively. Inhibition of PAX3-FOXO1 expression reduced cell proliferation and motility and allowed some myogenic differentiation [90]. In addition, comparative gene expression studies were performed identifying over 100 PAX3-FOXO1 gene targets. Cao *et al*. [76] performed ChIP sequencing studies using a PAX3-FOXO1-specific antibody and were able to identify 1,463 putative PAX3-FOXO1-binding sites in the human genome. Furthermore, PAX3-FOXO1-binding sites adjacent to MyoD, FGFR4 and IGF1R were verified as transcriptionally regulated by PAX3-FOXO1.

### *In vivo* models of ARMS

Many different transgenic and knock-in animal models have been attempted to recapitulate ARMS tumor formation *in vivo*. Several of these models have attempted to constitutively express PAX3/7-FOXO1 fusion proteins in the skeletal muscle lineage during development, only to result in developmental defects and not tumor formation [120-123]. Transgenic mice expressing PAX3-FOXO1 under the control of the PAX3 promoter and enhancer regions resulted in expression of PAX3-FOXO1 in the dorsal neural tube and lateral dermomyotome. PAX3-FOXO1 expression in this context appeared to interfere with normal PAX3 developmental functions including neural tube and neural crest abnormalities similar to those seen in PAX3 mutant Splotch mice. The majority of defects appeared to be in neural development, though defects were seen in hind limb skeletal muscle; however, no tumors developed [121,122].

Lagutina *et al*. [120] developed a model where PAX3-FOXO1 was knocked into the PAX3 locus. This knock-in locus expressed low amounts of PAX3-FOXO1, which in heterozygous pups was sufficient to result in developmental defects in the heart and diaphragm, leading to congestive heart failure and perinatal death, as well as malformations of some hypaxial muscles. However, neither chimeric adults nor their newborn heterozygous pups developed malignancies. It was hypothesized that PAX3-FOXO1 expression from the PAX3 control sequences was insufficient to cause ARMS formation, and downstream regions of the FOXO1 locus may be required to induce sufficient PAX3-FOXO1 expression to induce tumor development.

A PAX7-FOXO1 model of ARMS was also attempted in Drosophila [123]. Expression of UAS-hPAX7-FOXO1, under control of myosin heavy-chain Gal4, also resulted in developmental defects in the fly, evidenced by disorganized myogenic patterning. Though nothing resembling tumor formation was seen, this group did note dissemination and infiltration of non-native tissue by PAX7-FOXO1 expressing mononucleated cells, indicating an increase in invasive capacity of these cells.

Keller *et al*. [47] used a conditional PAX3-FOXO1 knock-in into the PAX3 locus, and Myf6-driven Cre expression. This allowed, upon Cre recombination, expression of PAX3-FOXO1 driven by the PAX3 promoter and 3’ FOXO1 genomic sequences that potentially contain cis-regulatory elements, a region absent from previous PAX3 knock-in strategies. This was the first animal model that successfully recapitulated the formation of ARMS, though at the low frequency of approximately 0.4% (1/228) and with latency of over 1 year (383 days). However, this frequency was greatly enhanced, and latency greatly reduced, in homozygote PAX3^P3Fa/P3Fa^ mice also lacking Trp53 or Ink4a/Arf. Subsequently, ARMS tumors have developed in this conditional PAX3-FOXO1 knock-in model with a *Pax7CreER* and *MCre* (Pax3 hypaxial muscle enhancer) also lacking functional Trp53 [124]. Moreover, histologically diagnosed fusion-negative ARMS tumors have been found to develop in conditional *Ptch1*^+/−^*Trp53*^−/−^ mice when Cre is expressed from *Pax7CreER*. The latency and incidence of ARMS tumor development in these different models have yet to be compared.

Clearly the problems that have arisen during the development of an animal model for ARMS indicate that the timing and the cell lineage targeted for PAX3-FOXO1 expression are very important for the development of ARMS tumor formation and for avoiding developmental defects. In a review [125] following the publication of the animal model [47], Keller *et al*. discuss the possibilities for the cell of origin for ARMS; because Keller *et al*. achieved the formation of ARMS tumors in their mouse model using Myf6-Cre-driven conditional PAX3-FOXO1, and Myf6 is usually expressed in differentiating skeletal muscle myotubes, they propose a potential dedifferentiation mechanism for ARMS development. However, the formation of a fusion gene such as PAX3-FOXO1 suggests that the cell of origin for ARMS should express both PAX3 and FOXO1 at the time that the translocation occurs, given that open chromatin is likely required for these two genomically distinct regions to translocate. Anecdotal evidence for this includes that the genome translocations that occur in many different cancer types occur between genes that are expressed in the cell type of origin. A recent study by Osborne *et al*. [126] showed that the MYC and IGH genes, which are involved in a chromosomal translocation common in Burkitt lymphoma, are colocalized at the same transcription factories more often in activated B-cells, the originating cell of Burkitt lymphoma, than resting B cells. This colocalization at the same transcription factory allows for close proximity of these gene loci in euchromatin, providing the circumstances where these genes would be in close association, facilitating the specific translocation event. PAX3 is rapidly downregulated upon myoblast differentiation, so it would be unlikely that the PAX3 loci would be expressed in a nascent myotube expressing Myf6, making it difficult to understand how translocation could occur in nascent myotubes and therefore cast some doubt on whether the dedifferentiation model is feasible. However, it is possible that Myf6 expression does rarely occur in a small subset of undifferentiated myogenic cells in conjunction with PAX3. This could allow for this model to produce ARMS tumors and account for the low frequency at which these tumors are seen as well as the requirement for homozygous PAX3-FOXO1 knock-in alleles [47].

From these animal models it is apparent that the timing of PAX3-FOXO1 expression is critical for ARMS development. Too early and widespread expression of PAX3-FOXO1 expression can result in developmental defects and no apparent tumor development [120-123], whereas later expression of PAX3-FOXO1, via a Myf6-driven Cre recombinase, does cause disease, though at a low frequency [47]. Perhaps inducible expression, driven by various myogenic genes with carefully characterized expression profiling, would result in an increased frequency of disease and help to narrow down the exact stage in which PAX3-FOXO1 expression drives ARMS tumorigenesis. Nevertheless, the cell of origin for ARMS is yet to be identified, and animal models of ARMS will no doubt play an important role in its identification.

## Conclusion

**Figure 2:**
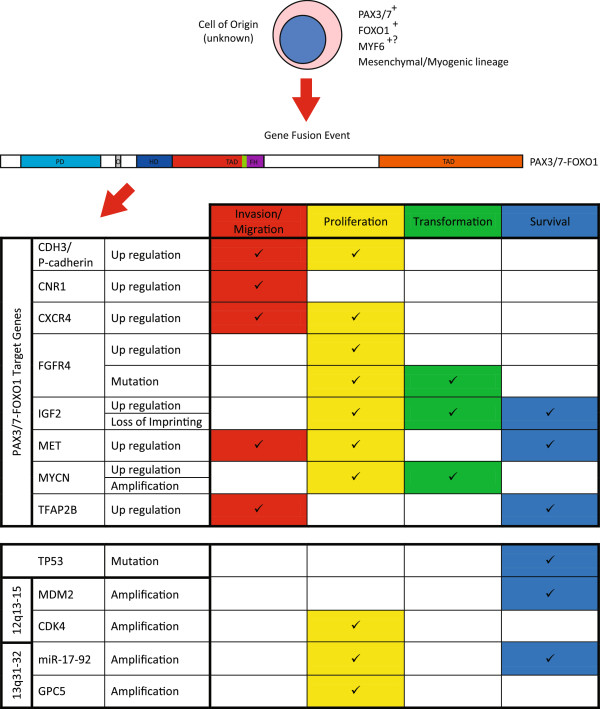
**Review summary: **Fusion gene regulated genes contributing to alveolar rhabdomyosarcoma. Rhabdomyosarcoma develops from an unknown cell of origin from the mesodermal lineage that may be skeletal muscle specified. This cell likely expresses both PAX3/7 and FOXO1 and may also express Myf6. A gene fusion event resulting in a PAX3/7 DNA-binding domain fused to a more potent transcriptional activation domain occurs. This fusion transcription factor is capable of inducing a group of PAX3-FOXO1-regulated genes that contribute to ARMS development in conjunction with other genetic lesions.

To date, numerous factors (outlined in Figure 2) have been identified that contribute to ARMS tumor development and its aggressive clinical phenotype. These consist of both PAX3/7-FOXO1 target genes, such as N-MYC, IGF2, MET, CXCR4, CNR1, TFAP2B, FGFR4 and P-cadherin, and PAX3/7-FOXO1 cooperating factors, such as the abrogation of the p53 pathway, IGF2 deregulation, N-MYC and miR17-92 amplification, and IRIZIO expression. Future ARMS research will continue to discover the mechanisms by which ARMS tumorigenesis occurs. This will involve the identification of more PAX3-FOXO1 target and cooperating genes; more importantly, the mechanisms by which these genes contribute to tumorigenesis will be elucidated. It is critical that we develop a mechanistic understanding of how these factors contribute and interact to perpetrate ARMS tumorigenesis. This will allow new opportunities to develop specifically targeted therapies for this aggressive pediatric disease.

## Abbreviations

ARMS: Alveolar rhabdomyosarcoma; bHLH: Basic helix loop helix domain; CDK: Cyclin-dependent kinase; CNR1: Cannabinoid receptor 1; ERMS: Embryonal rhabdomyosarcoma; FH: Forkhead DNA-binding domain; FKHR: Forkhead in rhabdomyosarcoma (now known as FOXO1); HD: Homeodomain DNA-binding domain; HGF/SF: Hepatocyte growth factor/scatter factor; LOH: Loss of heterozygosity; LOI: Loss of imprinting; MEF: Mouse embryonic fibroblast; miR: Micro RNA; MSC: Mesenchymal stem cells; PD: Paired box DNA-binding domain; PPTP: Pediatric Preclinical Testing Program; RMS: Rhabdomyosarcoma; SDF-1: Stromal-derived factor-1; SHH: Sonic hedgehog; siRNA: Short interfering RNA; TFAP2B: Transcription factor AP2 b.

## Competing interests

The authors have no competing interests to declare.

## Authors’ contributions

AM was responsible for the drafting of the manuscript. GG was responsible for critical revision of the content and approved the final version of the manuscript. All authors read and approved the final manuscript.
